# Heterologous Expression and Immunogenic Potential of the Most Abundant Phospholipase A_2_ from Coral Snake *Micrurus dumerilii* to Develop Antivenoms

**DOI:** 10.3390/toxins14120825

**Published:** 2022-11-24

**Authors:** Luz E. Romero-Giraldo, Sergio Pulido, Mario A. Berrío, María F. Flórez, Paola Rey-Suárez, Vitelbina Nuñez, Jaime A. Pereañez

**Affiliations:** 1 Research Group in Toxinology, Pharmaceutical, and Food Alternatives, Pharmaceutical and Food Sciences Faculty, University of Antioquia, Medellín 50010, Colombia; 2 Tropical Disease Study and Control Program—PECET, University of Antioquia, Medellín 50010, Colombia; 3 LifeFactors Zona Franca SAS, Rionegro 54047, Colombia; 4 Centro de Investigación en Recursos Naturales y Sustentabilidad, Universidad Bernardo O’Higgins, Santiago 8320000, Chile; 5 Microbiology School, University of Antioquia, Medellín 50010, Colombia

**Keywords:** recombinant protein, antibodies, *Micrurus dumerilii*, coral snake antivenoms, phospholipase A_2_

## Abstract

*Micrurus dumerilii* is a coral snake of clinic interest in Colombia. Its venom is mainly composed of phospholipases A_2_ being MdumPLA_2_ the most abundant protein. Nevertheless, *Micrurus* species produce a low quantity of venom, which makes it difficult to produce anticoral antivenoms. Therefore, in this work, we present the recombinant expression of MdumPLA_2_ to evaluate its biological activities and its immunogenic potential to produce antivenoms. For this, a genetic construct rMdumPLA_2_ was cloned into the pET28a vector and expressed heterologously in bacteria. His-rMdumPLA_2_ was extracted from inclusion bodies, refolded in vitro, and isolated using affinity and RP-HPLC chromatography. His-rMdumPLA_2_ was shown to have phospholipase A_2_ activity, a weak anticoagulant effect, and induced myonecrosis and edema. The anti-His-rMdumPLA_2_ antibodies produced in rabbits recognized native PLA_2_, the complete venom of *M. dumerilii*, and a phospholipase from another species of the *Micrurus* genus. Antibodies neutralized 100% of the in vitro phospholipase activity of the recombinant toxin and a moderate percentage of the myotoxic activity of *M. dumerilii* venom in mice. These results indicate that His-rMdumPLA_2_ could be used as an immunogen to improve anticoral antivenoms development. This work is the first report of an *M. dumerilii* functional recombinant PLA_2_.

## 1. Introduction

Coral snakes represent the Elapidae family in America; they are constituted by three genera *Micrurus, Leptomicrurus,* and *Micruroides* [1,2], although the phylogenetic findings do not separate *Leptomicrurus* from *Micrurus* in a concluding way [3]. *Micrurus* is the most abundant genus, with about 80 species distributed from the southern United States to Northern Argentina [4]. *M. dumerilii* is a species widely distributed in Colombia, covering the west, center, and north of the country; additionally, it is found in northwestern Ecuador, Northwestern Venezuela, and southeastern Panama. The species has an average length between 50 to 70 cm and presents a combination of three colors along the body, such as rings of red, white, and black [4].

*Micrurus* venoms are predominantly composed of Three-finger toxins (3FTxs) and Phospholipases A_2_ (PLA_2_s) [5]. The snake venom secretory PLA_2_ (svPLA_2_s) found in coral snake venom belongs to group IA. In addition, one venom can contain several isoforms of these enzymes [6]. The svPLA_2_s are a group of calcium-dependent enzymes that can hydrolyze glycerophospholipids at the middle ester bond (sn-2) [7]. According to Rey-Suárez et al. [8], the venom of *M. dumerilii* is mainly composed of PLA_2_s (52%).

In addition, it was identified that MdumPLA_2_ was the most abundant PLA_2_ from *M. dumerilii* venom [9]. It is an enzymatically active acid protein, without lethal effect, and with a high myotoxicity in mice.

The svPLA_2_s of coral snakes induce different pharmacological effects in victims such as neurotoxicity, myotoxicity, cytotoxicity, anticoagulant effects, cardiotoxicity, and edema, which are related to their capacity to hydrolyze membrane glycerophospholipids and multiple functional sites on its structure [10]. Despite this, the conservation of their structures and the broad spectrum of severe pharmacological effects suggest that svPLA_2_s represent a target for the production or enrichment of new antivenoms. In this way, the specific treatment for envenomation caused by snakebites is the intravenous administration of antivenoms produced from small doses of injected venom, mainly in horses [11]. Nevertheless, the *Micrurus* species produce a low quantity of venom and have serious issues surviving in captivity. In addition, they are difficult to find in the field, have ophiouphagus diets and semifossorial habits, and some species are relatively small [12,13].

It is known that antivenom production requires a high amount of venom to inject and produce an immune response in the animals selected [14,15]. Given the complexity of snake venoms, some of their components include toxins with different targets consequently resulting in diverse immunogenicity, thus generating antibodies targeting different antigens [16]. In addition, some toxins act in an independent manner, whereas others act synergistically, contributing to an increase in their toxicity [17,18]. Therefore, it makes it more challenging to direct the antibodies of the most significant therapeutic interest toward snake venom toxins [16]. Hence, getting high enough amounts of toxins from coral venom to produce antivenoms is a challenge that can be resolved by the recombinant expression of the target toxins into heterologous organisms. Although there are several systems to express recombinant proteins available, *Escherichia coli* is the first option due to its ease of genetic manipulation, inexpensive culture conditions, and rapid growth, among many other advantages, compared to eukaryotic expression systems and other bacteria [19].

In this context, Clement and colleagues [20] expressed a metalloprotease and a serineproteinase in a recombinant way in *E. coli* from mRNA transcripts of *Bothrops ammodytoides* venom gland. Moreover, the same author obtained heterologous expression and antibody recognition of a Mlat, a neurotoxin from the Mexican coral snake *Micrurus laticorallis* [21]. In parallel, Russo’s team’s [22] aim was to express two isoforms from the CB subunit from the crotoxin complex from *Crotalus durissus terrificus* venom, by using a prokaryotic system. They optimized and cloned the sequences into a plasmid vector (pG21a) and reached the expression of a biologically active recombinant protein. Moreover, Shimokawa-Falcão et al. [23] expressed Insularin, a disintegrin from the *Bothrops insularis,* using the expression vector SUMO Tag in *E. coli* strains.

Another important advance in this field was made by Wen-Li and colleagues [24], who achieved the functional expression and the characterization of a recombinant phospholipase A_2_ (PLA_2_) from sea snake *Lapemis hardwickii* as a soluble protein in *E. coli* using the vector pTRX. Furthermore, Guerrero-Garzón et al. [25] showed the heterologous expression of an α-neurotoxin (rD.H) from *Micrurus diastema* in a bacterial system. Starting at thirty different 3FTx transcript sequences obtained from the venom glands of four species of *Micrurus*, one transcript (D.H) encoded for short-chain α-neurotoxins was identified in the venom of *M. diastema*. Likewise, de la Rosa et al. [26] obtained a consensus sequence of an α-neurotoxin, using the sequences of species from elapid genera, including *Acanthophis*, *Oxyuranus*, *Walterinnesia*, *Naja*, *Dendroaspis*, and *Micrurus*. The consensus protein (ScNtx) was expressed in *E. coli* cells by cloning into the expression vector pQE30.

Among the several expression systems, the pET System is a powerful system designed to express recombinant proteins in *E. coli* BL21 strains upon the simple induction of the T7 RNA polymerase by IPTG [27]. In pET28a, target genes are cloned downstream of, and in-frame with, a poly-histidine purification tag (His6) under the control of the T7 promoter. Additionally, pET28a contains a lac operator to suppress uninduced expression. These features confer advantages that leverage its use for protein expression in prokaryotic heterologous systems.

This study presents the heterologous expression of the major PLA_2_ from *M. dumerilii*, its biological activities, immunologic recognition, and its potential to be used as an immunogen to produce antivenom formulations.

## 2. Results

### 2.1. Cloning Recombinant Toxin

**Figure 1:**
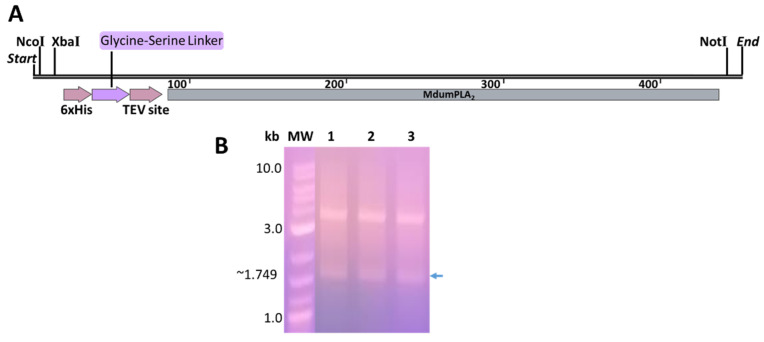
Plasmid carrying His-rMdumPLA_2_. (**A**) Genetic construction His-rMdumPLA_2_. (**B**) pET28a digestion test with XhoI and EcoRV in 1% agarose gel-stained ethidium bromide (Sigma, Saint Louis, MO, USA). MW: molecular weight marker (1 kb Plus DNA Ladder) (NEB); (1): His-rMdumPLA_2_ clone 1; (2): His-rMdumPLA_2_ clone 2; (3) His-rMdumPLA_2_ clone 3. The arrow indicates the fragment of 1749 bp with the expected size: target insert (451 bp) plus a fragment of 1411 bp (EcoRV-XhoI, coordinates 3797–5207) without 113 bp released in the cut. EcoRV cuts after position 158, and XhoI cuts after position 1573 on pET28a.

The genetic construct His-rMdumPLA_2_ of 451 bp was obtained (Figure 1A,B). The NcoI cleavage sites and NotI were included for the directional insertion into pET28a. Additionally, another XbaI cleavage site next to NcoI for further studies was also included. We added cytosine before the six-histidine segment to conserve the open reading frame. Proline and alanine were added before six amino acids corresponding to the linker glycine-serine, giving flexibility to the histidine tail. The cleavage site of the TEV protease was composed of seven amino acids (ENLYFQG) before the toxin gene was included. Finally, a stop codon UAG was included just after the end of the sequence target. In this context, the digestion of plasmid DNA and pET28a with NcoI and NotI allowed obtaining an insert of the expected size (451 bp) and a 5239 bp fragment corresponding to pET28a. The ligation between the insert and pET28a was evidenced by the growth of transformed colonies of *E. coli* DH5α in plates and inoculum of LB medium in the presence of ampicillin and kanamycin. The digestion test with XhoI (cut after the 158 position) and EcoRV (cut after the 1573 position) allowed to obtain a fragment of 1749 bp and another of 3958 bp (Figure 1B). These fragments matched with the expected sizes: the first matched with the target insert (451 bp) plus a fragment of 1411 bp (EcoRV-XhoI, coordinates 3797–5207) without 113 bp released by the cut with the endonucleases; the second matched with a fragment between XhoI-EcoRV (coordinates 5208–3796).

### 2.2. His-rMdumPLA_2_-BL21 (DE3) Expression and Isolation

**Figure 2:**
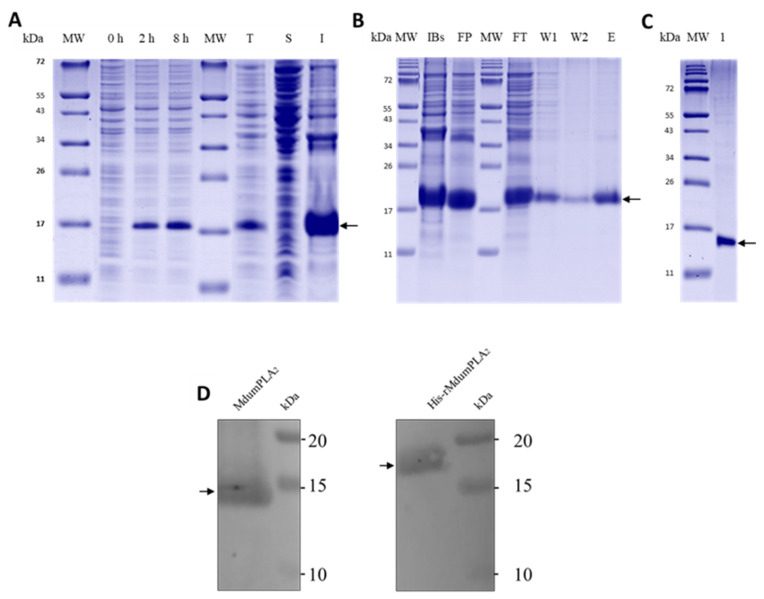
Expression of His-rMdumPLA_2_ in *E. coli* BL21 (DE3). A total of 10 µg of each sample were loaded in 14% Tris-Tricine SDS-PAGE gel and Coomassie G-250 stain. His-rMdumPLA_2_ molecular mass is about 17 kDa (arrow), according to molecular weight marker (11–250 kDa) (New England Biolabs, Ipswich, MA, USA). (**A**) His-rMdumPLA_2_ expression at three different times. MW: molecular weight markers (11–250 kDa), 0, 2 and 8 h. MW: molecular weight marker; T: total protein; S: soluble; I: insoluble. (**B**) Refolding and isolation by affinity chromatography. MW: molecular weight marker; IBs: solubilized inclusion bodies; FP: folded protein; MW: molecular weight marker; FT: Flowthrough; W1: Wash 1; W2: Wash 2; E: Elution. (**C**) TEV cut in 14% Tris-Tricine SDS-PAGE gel and Coomassie G-250 stain. A total of 10 µg of rMdumPLA_2_ was loaded and detected as a band of approximately 14 kDa. (**D**) Western blot using the anti-MdumPLA_2_ native antibody [1:100] coupled to peroxidase, obtained from rabbit inoculated with MdumPLA_2_ isolated from *M. dumerilii* venom by RP-HPLC. MdumPLA_2_ molecular mass is about 13 kDa (arrow in the left panel) and His-rMdumPLA_2_ molecular mass is about 17 kDa (arrow in the right panel). Precision Plus Protein Kaleidoscope Standard (10–250 kDa) (Sigma, Saint Louis, MO, USA) was used as molecular weight marker.

The expressed His-rMdumPLA_2_ recombinant protein in *E. coli* strain BL21 (DE3) was detected by SDS-PAGE (Figure 2A) and later by Western blot (Figure 2D) in an insoluble form (I) as inclusion bodies with a total yield of 10.1 mg per liter of culture medium after purification. After the TEV cut, a band was obtained in the expected rMdumPLA_2_ mass (about 14 kDa) (Figure 2C), which was close to the reported (~13.3 kDa) [8]. The molecular mass of His-rMdumPLA_2_ detected by SDS-PAGE was about 17 kDa (Figure 2A,B), close to the theoretical molecular mass (16,037 Da). In addition, the experimental molecular mass established by mass spectrometry was 15,810 Da.

**Figure 3:**
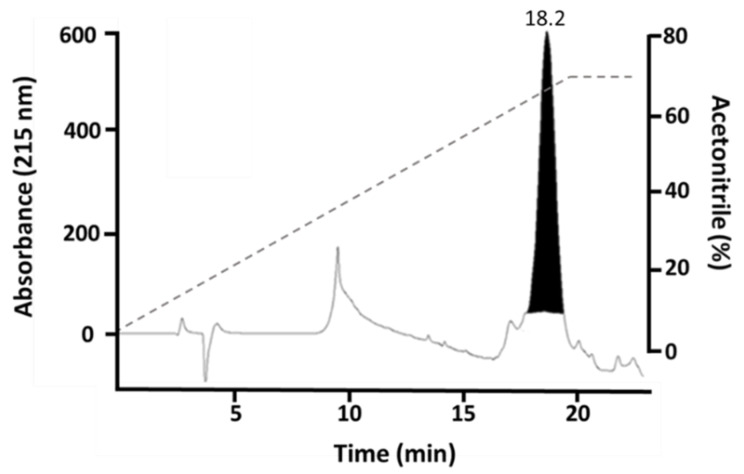
His-rMdumPLA_2_ purification by RP-HPLC chromatography. His-rMdumPLA_2_ purification by RP-HPLC chromatography on a C18 column (250 × 10 mm) eluted at 1 mL/min with an acetonitrile linear gradient. His-rMdumPLA_2_ was collected in the peak that eluted at 18.2 min.

Figure 2A shows the SDS-PAGE of proteins before and after expression in the *E. coli* strain BL21 (DE3). *E. coli* cells grew when the OD_600 nm_ was between 0.6–0.7. This time was considered as 0 h, just before induction. After IPTG induction in *E. coli* strain BL21 (DE3), a band with a molecular mass of about 17 kDa was obtained starting from 2 h until 8 h, with a slight gradual increment in its width. The insoluble (I) fraction obtained after cell lysate showed the presence of a thick band, whereas the soluble (S) fraction evidenced an irregular band. Figure 2B shows the solubilized inclusion bodies (IBs) and the folded His-rMdumPLA_2_ protein (FP). Using nickel affinity chromatography, one fraction corresponding to each stage of the process was obtained. In the four fractions (flowthrough, wash 1, wash 2, and elution), a band was obtained with His-rMdumPLA_2_ mass of about 17 kDa (Figure 2B), and its identity was confirmed by western-blot assay (Figure 2D). Verification of the molecular mass of rMdumPLA_2_ (~14 kDa) is shown in Figure 2C. About the isolation of the recombinant toxin by RP-HPLC, under the conditions described in the Materials and Methods section, the recombinant rMdumPLA_2_ fraction (Figure 3) was identified at an elution time of approximately 18.2 min.

### 2.3. Biological Activities

**Figure 4:**
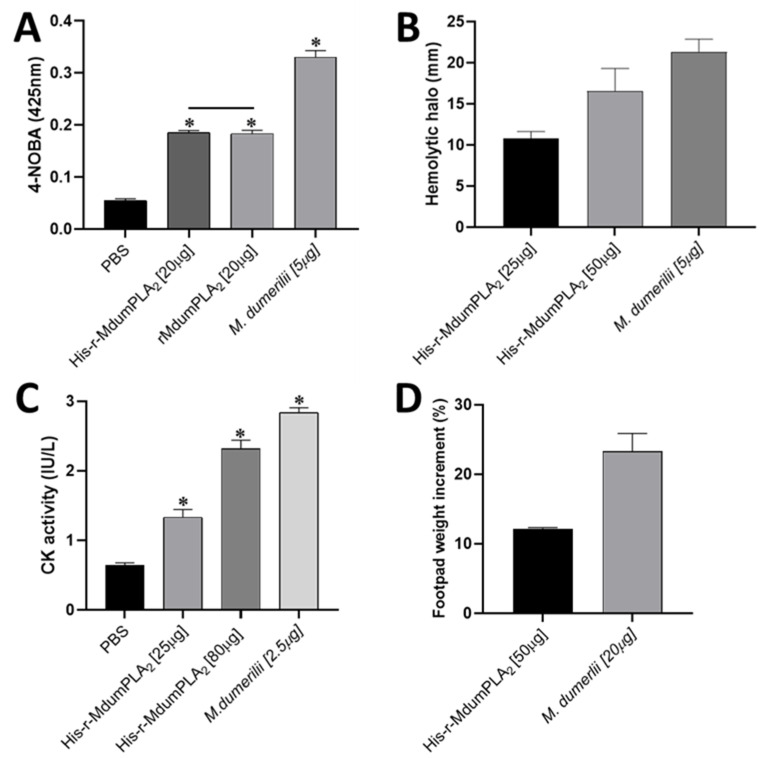
Biological activities of His-rMdumPLA_2_. (**A**) PLA_2_ activity by hydrolysis of substrate 4-NOBA. (**B**) PLA_2_ activity by indirect hemolysis. The bars show the diameter of the hemolytic halo in millimeters. The diameter of the hemolytic halo of negative control (PBS) was zero. (**C**) Myotoxic activity. The CK activity was determined from mouse plasma by kinetic assay, and the absorbance was recorded at 340 nm. (**D**) Edema-inducing activity. Edema was estimated by the percentage increment in the weight of the footpad with respect to the negative control (SS). * Indicates statistically significant differences with corresponding negative control (*p* < 0.05). The data show the mean ± SD (*n* = 3).

Both rMdumPLA_2_ and His-rMdumPLA_2_ were enzymatically active, evidenced by their ability to hydrolyze 4-NOBA compared with the negative control (PBS). However, there were no significant differences between rMdumPLA_2_ and His-rMdumPLA_2_ (Figure 4A). In the same way, His-rMdumPLA_2_ demonstrated an indirect hemolytic effect, in a dose-dependent manner (Figure 4A). Further, His-rMdumPLA_2_ increased the clot formation time about four-fold (221 min) in comparison to that of the negative control (PBS) (58 min). On the other hand, the recombinant toxin induced an increment in CK activity in a dose-dependent way (Figure 4B) when compared to the negative control (*p* < 0.0001). Moreover, His-rMdumPLA_2_ induced mild edema-forming activity. His-rMdumPLA_2_ induced myotoxic, enzyme, edema-forming, and anticoagulant activities, but it required higher doses than those used in the venom. In contrast, His-rMdumPLA_2_ did not cause lethality in mice of 18–20 g of body weight.

### 2.4. Anti-His-rMdumPLA_2_ Titers in Serum and Cross Immunological Recognition

**Figure 5:**
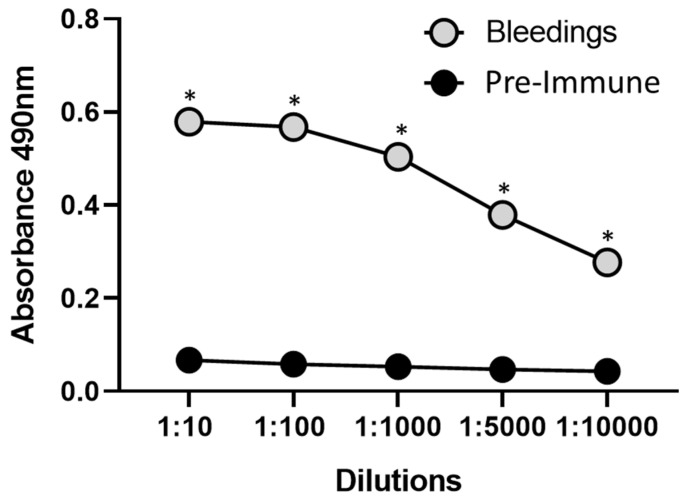
The titration curve of average antibodies in serum from four bleedings by ELISA against His-rMdumPLA_2_. A 96-well plate was coated with His-rMdumPLA_2,_ and serum from four bleedings was used in dilutions from 1:10 to 1:10,000. A peroxidase-labeled anti-rabbit IgG conjugate detected bound antibodies. The absorbance of antibodies anti-His-rMdumPLA_2_ is shown in the function of the dilution. * Indicates statistically significant differences with the pre-immune serum. The data show the mean ± SD (*n* = 3).

**Figure 6:**
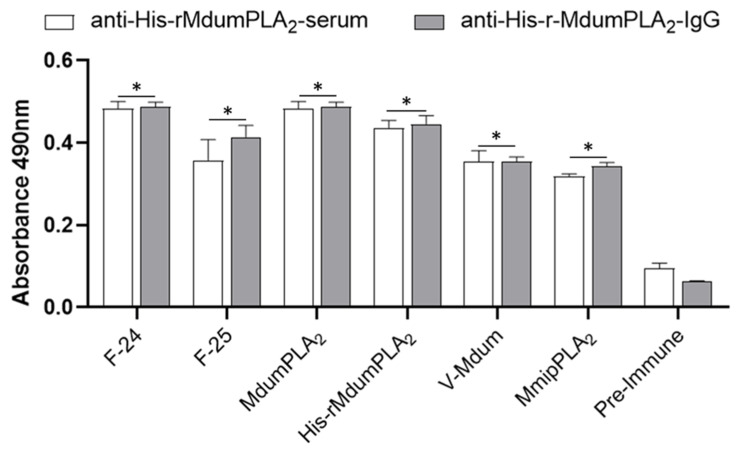
Immunoreactivity of serum and IgG anti-His-rMdumPLA_2_ against *M. dumerilii* venom (V-Mdum), His-rMdumPLA_2_, MdumPLA_2_, the native 24 and 25 fractions (F-24, F-25) close to MdumPLA_2_ in the PLA_2_ region from *M. dumerilii* proteome, and MmipPLA_2_ by ELISA. * Indicates statistically significant differences with the pre-immune serum (*p* < 0.0001). The data show the mean ± SD (*n* = 3).

An increase in antibody titers was observed against His-rMdumPLA_2_ until 1:10,000 (Figure 5), and significant statistical differences were detected when compared with pre-immune serum (*p* < 0.0001). Additionally, the serum and IgG anti-His-rMdumPLA_2_ recognized the His-rMdumPLA_2_, the venom *M. dumerilii*, native MdumPLA_2_, the native fractions (F24 and F25, fractions from PLA_2_ region from *M. dumerilii* proteome), and MmipPLA_2_ (a PLA_2_ from *M. mipartitus*) (Figure 6).

### 2.5. Neutralization of the Biological Activities of M. dumerilii Venom by Anti-His-rMdumPLA_2_-IgG

**Figure 7:**
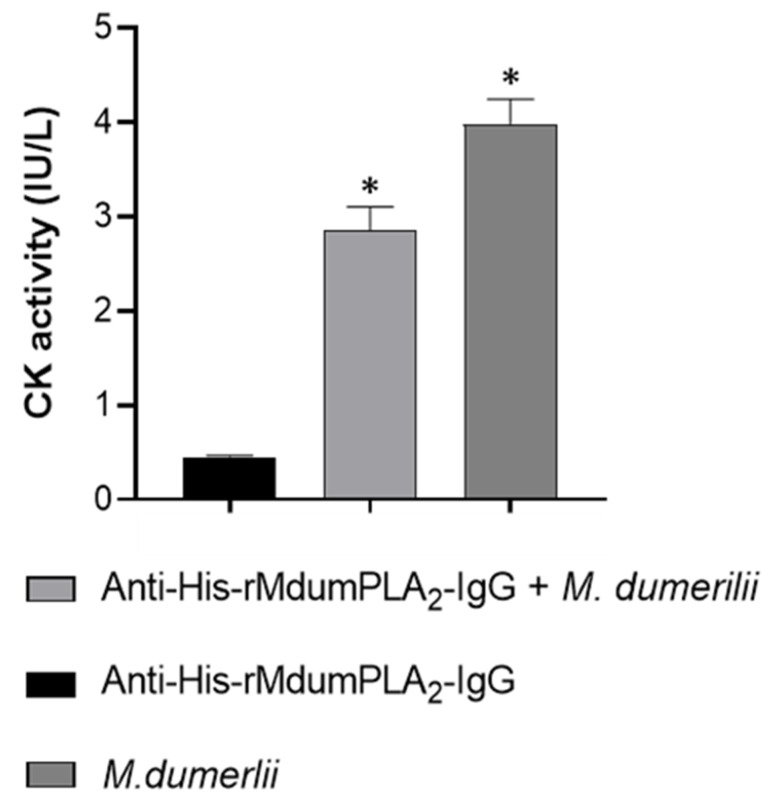
Neutralization of the myotoxic activity of *M. dumerilii* venom by anti-His-rMdumPLA_2_-IgG. 3 mg IgG anti-His-rMdumPLA_2_ mixed with 5 µg *M. dumerilii* venom was injected by the intramuscular route in mice, and CK activity was determined in serum. * Indicates statistically significant differences with anti-His-rMdumPLA_2_-IgG (*p* = 0.0005). The data show the mean ± SD (*n* = 3).

Anti-His-rMdumPLA_2_-IgG neutralized 100% the PLA_2_ activity of His-rMdumPLA_2_. Additionally, it neutralized the myotoxicity induced by *M. dumerilii* venom by about 28% (Figure 7). However, anti-His-rMdumPLA_2_-IgG did not neutralize the lethal effect of the *M. dumerilii* whole venom.

## 3. Discussion

Snake venoms are complex mixtures of proteins, enzymes, peptides, non-enzymatic proteins, and inorganic components. These are rich sources of active compounds with a broad spectrum of biological activities [28]. Indeed, several amino acid sequences and crystal structures of many snake venom PLA_2_-isoforms have been reported, showing high identities and a conserved structural scaffold [29,30], but their biological effects differ [31].

The PLA_2_ are enzymes that occur in almost all venoms, including *Micrurus* genus, and they play a vital role in the pharmacological effects observed in snakebites. The most abundant PLA_2_ of *M. dumerilii* was isolated and sequenced by Rey-Suarez et al. [8]. This toxin induced myotoxicity and edema, but it was not lethal. This study produced it in a recombinant form and its potential to develop antivenoms was tested.

*E. coli* is a recombinant system that offers significant advantages such as the knowledge of its genetics, plasmids availability, a variety of inducible promoters, and low production costs [32]. For this reason, a genetic construct that would allow overexpressing a protein in a prokaryote expression model was developed, using the optimization of sequences during the construct’s design process to increase its expression levels per se [33].

Among the different procaryotic expression systems, one of the advantages of using pET vectors is their medium copy number, which makes it an efficient system to produce a large amount of the target protein in the competent cells of *E. coli*. In addition, transcription of the inserted gene achieves when pET28a was inserted in BL21 (DE3), which carries the gene for T7 RNA polymerase. When induced, this gene plays a selective role, resulting in almost all the cell’s resources being focused on targeting gene expression [34]. In this sense, after purification, the yield (10.1 mg/L) of His-rMdumPLA_2_ showed a medium expression level, considering recovery yields of the recombinant protein in a range of 10–50 mg/L [35,36]. It is important to describe that pET, and other expression vectors have intrinsic features that can delimit the yield of the heterologous protein [37]. Moreover, *E. coli* strains express differentially heterologous genes because of specific genomic characteristics that can be a decisive factor in the high expression of recombinant proteins [32].

Understanding the above, when we compare our results with other studies, the yield (10.1 mg/L) of His-rMdumPLA_2_ after purification was superior to that of Wen-Li et al. [24], who obtained a final yield of approximately 2.5 mg/L of pure rPLA_2_-9 using the strain BL21 (DE3) but different expression vector. In the same way, our results contrast with those reported by Clement et al. [20] and Guerrero-Garzón et al. [25], who used the plasmid pQE30 and expressed in the strains *E. coli* Origami, the recombinants rBamSP_1 (1 mg/L) and rBamMP_1 (1.8 mg/L), and the recombinant rD.H. (4.2 mg/L) after purification, respectively. Other researchers like Shimokawa-Falcão et al. [23] obtained a yield of LgRec2 of about 24 mg/L of purified protein and 20 mg/L of the disintegrin Insularin. Both recombinant proteins were cloned on the pSUMOUlp1 vector and expressed in BL21 Star™ (DE3) cells *E. coli*.

According to Woestenenk et al. [38], high protein production levels could be mainly due to a high growth rate of the host cells, in this case, to a balanced exponential growth phase of BL21 (DE3) *E. coli*. This growth, in turn, depends significantly on the overexpressed target protein. However, the essential factor in producing recombinant protein is the absolute amount of soluble protein per culture volume rather than the fraction of soluble protein per total protein. Even though the concentration soluble fraction is high, that does not mean high protein yields after purification. Additionally, high host cell growth levels combined with vector encoding N-His tag allowed total protein purification. Mohanty and colleagues [39] have also postulated that the tag could confer more stability to the protein. Fundamentally, the sequences used in the design of genetic construct, the strategy of cloning, or the vector backbones should be considered when expressing the recombinant protein.

It is important to note that although His-rMdumPLA_2_ is a cysteine-rich protein with seven disulfide bonds, which confer enzyme stability [40], it was expressed as IBs. Thus, it was necessary to solubilize it with a denaturing agent. As the refolding process is a conformational change from the unfolded to the native protein state [41], it implies a balance between aggregation and solubilization for achieving an active protein. However, it is known that during the refolding process can occur intermolecular and intramolecular interactions [42], that may lead to a non-native structure which, in turn, may result in misfolding with the formation of non-native disulfides bonds [41] and thus, in an incomplete folding [43,44]. Furthermore, impurities present in solubilized IBs can associate with the expressed protein and in some way interfere with its refolding [42]. In this case, His-rMdumPLA_2_ was able to adopt a stable soluble conformation but the exact architecture of the active site could be deficient; therefore, comparable activity with native protein was not obtained. Anyway, the solubilization of the His-rMdumPLA_2_ IBs allowed a sufficiently flexible and disordered structure of protein that conferred biological activity with respect to native protein or the complete venom. In fact, in some proteins, it has been reported that IBs solubilization allows recovery of 50% or less of the bioactive product [45] and no biologically active product in other cases [46].

The mass of His-rMdumPLA_2_ detected in SDS-PAGE was very close to the theoretical molecular mass of His-rMdumPLA_2_ (~16 kDa). However, it differed from the native MdumPLA_2_ which has a molecular mass of 13,288 Da [8]. This difference was due to the 6His-tag, sequences coding to linker GS and the excision site of protease TEV. This was demonstrated when cutting the His tail using protease TEV, since the obtained molecular mass was comparable to the native MdumPLA_2_.

His-rMdumPLA_2_ was enzymatically active, as shown by its ability to hydrolyze the 4-NOBA synthetic substrate, and indirect hemolytic activity. In both cases, it did not display significant differences when we compared the enzymatic activity rMdumPLA_2_. This result allowed us to decide to continue using His-rMdumPLA_2_ since when the His tail is cut with the TEV protease, some amounts of the recombinant protein are lost, which results in a decrease in the quantity of rMdumPLA_2_ available for the rest of the assays.

In the same way, His-rMdumPLA_2_ induced a dose-dependent increase of plasma CK activity after (i.m.) injection into the gastrocnemius muscle of mice. Herein, the forming of incorrectly folded proteins and insoluble aggregates in the reducing environment of bacterial cytoplasm, and low recovery of the bioactive product might be related to a decreased myotoxic effect. Furthermore, the complex process of refolding protein in the presence of the 6His tag may cause differences in structural motifs. However, this subject requires further studies.

PLA_2_s trigger the synthesis of lipid regulatory molecules at the inflammation site. These molecules are fatty acids, their derivatives (prostaglandins, leukotrienes, and thromboxanes), and phospholipid platelet-activating factor (PAF) [29]. Considering that PLA_2_s are venom components implicated either directly or indirectly in the induction of edema [47,48], we found that His-rMdumPLA_2_ induced weak edema in the mouse footpad. Low recovery of the bioactive product from solubilized IBs, even with a medium level of expression of recombinant protein, as mentioned above may affect edematogenic activity. Nevertheless, this finding could be important from the point of view of animal welfare because it contributes to maximizing the host antibody response and minimizing the inflammatory lesions associated with the nature of the antigen [49]. 

According to anticoagulant abilities, PLA_2_ enzymes may be strong, weak, and non-anticoagulant enzymes [50]. His-rMdumPLA_2_ had a weak anticoagulant effect because it showed this activity in doses higher than 15 μg/mL [50]. Nevertheless, the weaker anticoagulant activity of His-rMdumPLA_2_ might be explained by possible structural differences to native PLA_2_, especially in anticoagulant region (pharmacological site) that binds to a coagulation Factor Xa and inactivates it [51]. However, these discrepancies were not tested in this study, thus, they should be addressed in further studies.

In general, we obtained a functional His-rMdumPLA_2_ protein. Nevertheless, its bioactivity was different in comparison to native PLA_2_ [8]. These results might be due, in part, to misfolding of protein, given that during the refolding process, the presence of the His tag may cause negative effects on the tertiary structure or in the biological activity of the chimeric protein, such as it was reported by Khan et al. for MSP1(42) protein [52]. Nevertheless, those structural changes may not affect the active site, interfacial binding surface and hydrophobic channel, which are involved in the catalytic mechanism of PLA_2_s [31], but this hypothesis should be addressed in further studies. Despite that, rabbit polyclonal antibodies generated against His-rMdumPLA_2_ recognized both *M. dumerlii* venom, native Mdum-PLA_2_, the 24 and 25 fractions of the phospholipases region from the proteome of *M. dumerilii* and MmipPLA_2_. It is possible that some conformational epitopes have been recognized by some of anti-His-rMdumPLA_2_ antibodies generated by the rabbit’s immune system.

It is important to note that the pattern of the group I PLA_2_s from elapid venoms is conserved between MmipPLA_2_ and MdumPLA_2_ according to the study of Rey-Suarez et al. [8], and furthermore when the amino acid sequences of both toxins were compared, they shared 65% identity [8]. In this regard, and in concordance with the mentioned above, immunological cross recognition of antibodies against His-rMdumPLA_2_ might be the result of a conserved structural scaffold of both MdumPLA_2_ and MmipPLA_2_.

On the other hand, anti-His-rMdumPLA_2_-IgG did not neutralize the lethal activity of the *M. dumerilii* venom; moreover, these antibodies neutralized 100% of the PLA_2_ activity of itself and a moderated percentage of the myotoxicity activity of *M. dumerilii* venom. These results could be explained by the presence of other lethal components or other strongly toxic PLA_2_ in *M. dumerilii* venom. It is known that toxins and other components of snake venom can act independently or synergistically, to cause a broad spectrum of toxic effects such as local or systemic damage [53]. In addition, due to the presence of the “pharmacological sites” in the three-dimensional structure of the PLA_2_s, neutralizing catalytic activity does not ensure the inhibition of the biological effects, such as myotoxicity or edema-forming [30]. Likewise, it has been reported that low molecular mass proteins, such as PLA_2_ generate a low immune response compared to high molecular mass proteins [5,54,55]. Thus, it is to be expected that MdumPLA_2_ or the whole *M. dumerilii* venom was not neutralized by anti-His-rMdumPLA_2_-IgG. Unfortunately, because we did not have enough anti-His-rMdumPLA_2_-IgG, it was impossible to test other doses or carry out more assays.

The use of most abundant toxins involved in envenoming not only of *M. dumerilii* (in this case) but of different coral snake species with clinical relevance [56], and whose availability is reduced by other factors such as survival captivity and a low venom production [12], is key to antivenom development. The results of His-rMdumPLA_2_ suggest that this recombinant toxin could be included in the mix of venoms and other toxins used to produce antivenoms against different species of elapids.

## 4. Conclusions

We demonstrate the heterologous expression and refolding of a functional version of a PLA_2_ of *M. dumerilii* (His-rMdumPLA_2_), which is the most abundant PLA_2_ from *M. dumerilii* venom (MdumPLA_2_). Additionally, this finding is the first report of a recombinant PLA_2_ from *M. dumerilii* venom. Moreover, we demonstrate cross-recognition of His-rMdumPLA_2_ with the native PLA_2_, the *M. dumerilii* whole venom, and one PLA_2_ of another specie of the *Micrurus* genus. Our findings allow us to conclude that antibodies anti-His-rMdumPLA_2_ neutralized the PLA_2_ activity of the same recombinant toxin and moderately neutralized the myotoxic effect produced by the *M. dumerilii* venom in mice. We highlight the importance of improving the production methods of the recombinant PLA_2,_ intending to boost the neutralizing capacity of immune sera induced by recombinant products and therefore increase the clinical efficacy of the antivenoms. Finally, it is fundamental to continue exploring the expression of recombinant versions of elapid toxins as an innovative source of venom-like resources to antivenom production.

## 5. Materials and Methods

### 5.1. Materials

Restriction enzymes NcoI, XbaI, NotI, XhoI and EcoRV, and T4 DNA ligase were purchased from New England Biolabs (NEB) (Ipswich, MA, USA). *Escherichia coli* DH5α (Invitrogen) strain was used for gene cloning and plasmid propagation meanwhile *E. coli* strain BL21(DE3) (Stratagene, San Diego, CA, USA) was used for protein expression. The plasmid pET28a (Novogene, Cambridge, UK) was used as an episomal vector to produce the fusion construct as described below.

### 5.2. Venoms and Animals

*M. dumerilii* venom (lyophilized) was provided by the Serpentarium of the University of Antioquia and obtained from adult specimens collected in Antioquia, Colombia. Swiss-Webster mice of both sexes with 18–20 g of body weight were utilized in all in vivo experiments under the ethics committee of Antioquia University (license No. 110 of 2017). New Zealand white rabbits (2.15 kg) were utilized in vivo immunizations experiments according to the ethics committee above.

### 5.3. Optimization and Genetic Construction

The amino acidic sequence C0HKB8 of phospholipase A_2_ (MdumPLA_2_) of *M. dumerilii* from the database UniProt “https://www.uniprot.org (accessed on 10 May 2019)”was loaded in the OPTIMIZER tool “http://genomes.urv.es/OPTIMIZER/ (accessed on 20 September 2019)”, using the preferential codon usage according to Kazusa database “http://www.kazusa.or.jp/codon/ (accessed on 20 September 2019)”. The open reading frame (ORF) of the optimized sequence was verified by in silico translation using the ExPASy tool “https://www.expasy.org/ (accessed on 20 September 2019)”. Finally, the optimized sequence was fused with an N-terminal polyhistidine tag (6HisTag) followed by a glycine-serine linker and a TEV protease recognition site, right in front of the Met-ini of the PLA_2_ ORF. For cloning into the MCS pET28a, we set a 5′ NcoI site in front of the 6HisTag at the 5′ end and a 3′ NotI site downstream of the stop codon. The construct synthesis was made by General Biosystems and delivered in the commercial cloning vector pUC57_BsaI_Free. A short name HisrMdumPLA_2_ was assigned to the plasmid product (Figure 1A).

### 5.4. Cloning His-rMdumPLA_2_

First, the plasmid pUC57-His-rMdumPLA_2_ was propagated in competent cells of *E. coli* strain DH5α by standard heat shock transformation and selection in LB media containing 100 μg/mL of ampicillin. Three clones were selected and grown overnight in LB medium with ampicillin. The plasmid DNA of each clone was recovered using FavorPrep Plasmid Extraction Mini Kit (FAVORGEN Biotech Corporation). The integrity of the plasmid DNA was assessed by 1% agarose electrophoresis and quantified using the Thermo Scientific™ NanoDrop 2000 from Thermo Scientific (Waltham, MA, USA). The same procedure was applied to the empty pET28 vector using kanamycin as the selection antibiotic. The plasmid DNA and the pET28a expression vector were digested with specific restriction enzymes NcoI and NotI in the 5′ and 3′ positions, respectively. The digestion reaction (50 µL) was incubated for 3 h at 37 °C and the digestion products were analyzed on a 1% agarose gel. The bands with the expected size for the insert and linearized vector were cut from the gel and purified using the GeneJet Plasmid kit from Thermo Scientific, following the manufacturer’s instructions. For the insertion of the construct into pET28a, a ligation reaction (20 µL) was set using a 3:1 excess of insert and T4 DNA ligase. The reaction was incubated at room temperature for 2 h. A total of 5 µL of the ligation reaction was transformed in DH5α cells and incubated overnight in selection LB media containing 50 μg/mL kanamycin. Final propagation and recovery were made as described above. Clones and the directionality of the insert were checked by XhoI and EcoRV digestion and agarose gel analysis. EcoRV cuts after position 158, and XhoI cuts after position 1573 on pET28a.

### 5.5. Expression of pET28a-His-rMdumPLA_2_

As mentioned above, one clone of the recombinant plasmid pET28a-His-rMdumPLA_2_ was used to transform BL21 (DE3) cells by heat shock. Transformed cells were selected in LB-Kanamycin [50 mg/mL]. One single colony was used for propagation and protein induction each time. For protein expression, 1 L of LB medium was enriched with 5 mL of the overnight pre-culture. Then, the culture flask was grown at 37 °C and 180 rpm until it reached OD 600 nm = 0.6–0.7, and protein expression was induced with 0.5 mM IPTG (isopropyl-β-D-thiogalactopyranoside) at 37 °C for eight hours. Cells were harvested by centrifugation at 32,000× *g* at 4 °C for 30 min. The cell pellet was washed with PBS 1X buffer pH 7.4 and centrifuged at 13,000× *g* for 30 min at 4 °C. Finally, the cell pellet was stored at −20 °C until use or until six months as the maximum storage period.

### 5.6. Protein Purification and Refolding

The protocol mentioned above delivered HisrMdumPLA_2_ chimeric protein as inclusion bodies which were purified as follows. The cell pellet was thawed on ice and resuspended in lysis buffer [100 mM Tris pH 8.5, 10 mM EDTA] and sonicated 7 min, 2 sec ON, and 2 sec OFF at 22 °C and 20% power in Ultrasonic Cell Disruptor (BIOBASE Biodustry, Jinan, Shandong, China). The lysate was centrifuged at 32,000× *g* at 4 °C for 30 min. The insoluble fraction or inclusion bodies were dissolved in solubilization buffer (100 mM Tris-HCl pH 8.0, 10 mM EDTA, 8 M urea) at 180 rpm, at room temperature overnight. The solubilized protein was clarified by centrifugation at 32,000× *g* at 4 °C for 30 min, and the supernatant was transferred to a SPECTRA/Por MWCO: 3.5 kDa tubbing membrane. Refolding of the denatured His-rMdumPLA_2_ was achieved by stepwise removal of the urea by dialysis against refolding buffer (200 mM Tris pH 8.5, 10 mM EDTA) containing reduced concentrations of urea from 4 M to 0 M, making exchanges of the refolding buffer every 12 h in constant agitation (110 rpm) at 4 °C. Once urea 0 M concentration was reached, the protein was centrifuged at 32,000× *g* at 4 °C for 30 min and the supernatant or the recombinant solubilized protein was recovered.

### 5.7. Purification of His-rMdumPLA_2_

The first purification step of His-rMdumPLA_2_ was performed by affinity chromatography using Ni-NTA agarose resin. The recombinant refolded fraction protein was applied twice to a 2 mL bed of resin (Qiagen™ Ni-NTA Superflow) by gravity flow previously equilibrated with refolding buffer without urea [20 mM Tris pH 8.5, 100 mM NaCl]. Next, the first wash with equilibrium buffer was performed. Then, two washes with 20 mM imidazole and 500 mM NaCl were applied each time to eliminate cellular debris and nonspecific proteins. Finally, a 360 mM imidazole buffer was applied to elution the target protein. The concentration of protein (in mg/mL) was determined by the Bradford protein assay [57] using the equation *y* = 0.6625*x* − 0.0535 with an R-squared value of 0.9978, where *y* is absorbance and *x* is concentration. In addition, only to check the molecular mass approximate of rMdumPLA_2_, we cut the 6His-Tag with [10 mg/mL] TEV protease by dialysis, using buffer [20 mM Tris pH 8.5, 100 mM NaCl, 5 mM 2-Mercaptoethanol (Sigma, Saint Louis, MO, USA)] at pH 8.5 and a SPECTRA/Por MWCO: 3.5 kDa tubbing membrane. The TEV cut was analyzed with 14% Tris-Tricine SDS-PAGE gel, described by Laemmli [58] with modifications of Schägger and Gebhard [59] under reducing conditions. Subsequently, in a second purification step of His-rMdumPLA_2_, the sample was centrifuged and applied to reverse-phase high-performance liquid chromatography (RP-HPLC) C18 column (250 × 10 mm, 5 µm particle: Restek, Bellefonte, PA, USA), using a Shimadzu Prominence-20A chromatograph. Elution was performed at 1 mL/min by applying solution B (acetonitrile + 0.1% TFA) as lineal gradient 0 to 70% B over 35 min. The elution profile was monitored at 215 nm in a UV/VIS photodiode array detector (Shimadzu, Kyoto, Japan). The sample was vacuum dried in a Vacufuge Plus Complete System (Eppendorf, Hamburg, Germany) at 30 °C and resuspended into Milli-Q water to be analyzed by 14% Tris-Tricine SDS-PAGE and to confirm its molecular mass by mass spectrometry. The heterologous expression of His-rMdumPLA_2_ was confirmed by western-blot assay following the protocol of Lomonte et al. [55]. An anti-MdumPLA_2_ native antibody coupled to peroxidase (obtained from rabbit inoculated with MdumPLA_2_), was used for this. Additionally, a nitrocellulose membrane (0.45 mm) in a TRANS-BLOT SD (BIO-RAD, California, United States) system, and a transference buffer (192 mM Glycine, 25 mM Tris, 20% Methanol, and pH 8.3) were used. Membrane blockade was performed with 1% BSA/1% casein and the washes with 1:10 dilution of the blocking solution. Color development was performed using 6.5 mM 4-Cl-1 naphthol in 0.02 M Tris buffer at pH 7.5 in the presence of H_2_O_2_.

### 5.8. Mass Spectrometry

Intact mass of the His-rMdumPLA_2_ was determined by direct infusion ESI-MS in a Q-Exactive Plus^®^ instrument (Thermo Scientific, Waltham, MA, USA). Proteins were dissolved in 50% acetonitrile/water containing 0.1% formic acid at 50–100 μg/mL and infused at 5 μL/min into a HESI source, to acquire a full MS scan in the 800–2000 *m*/*z* range, at resolution of 140,000 (at *m*/*z* 240), in positive mode. The acquired MS spectra of the multiply charged ion series were deconvoluted using Freestyle^®^ v.1.5 (Thermo, Scientific, Waltham, MA, USA) to obtain monoisotopic masses [60].

### 5.9. Myotoxic Activity

Groups of three mice were injected intramuscular (i.m.) route with 50 µg and 80 µg of recombinant toxin, and another group was 2.5 µg *M. dumerilii* venom diluted in 100 µL of saline solution (SS). A control group received 100 µL of SS alone. At 3 h, the animals were caudal veins bleeding and blood was collected into heparinized capillary tubes, and plasma creatine kinase (CK; EC 2.7.3.2) activity was determined by a kinetic assay (CK-NAC UV AA Kit) from Weiner Lab (Boston, MA, USA). Triplicate measurements were performed on each sample in a UV-1700 PharmaSpec Spectrophotometer (Shimadzu, Kyoto, Japan) and CK activity was expressed in U/L.

### 5.10. Edema-Forming Activity

Groups of three mice received a subcutaneous (s.c.) injection of 50 µg of recombinant toxin or 20 µg *M. dumerilii* venom dissolved in 50 µL SS, into the right footpad. A similar volume of SS was injected into the left footpad in both groups as a negative control. After 2 h, mice were sacrificed by isoflurane inhalation, both feet were cut and weighed, as described by Gutierrez et al. [61], and the percentage increment in weight with respect to negative control was determined.

### 5.11. PLA_2_ Activity

PLA_2_ activity of His-rMdumPLA_2_ and rMdumPLA_2_ was evaluated in vitro using synthetic monodisperse substrate 4-NOBA (4-nitro-3-octanoyloxy-benzoic acid) [62] for 96-well plates. For this, 20 µg of the recombinant toxin dissolved in 10 mM Tris pH 8.0, 0.1 M NaCl, and 10 mM CaCl_2_ buffer in presence of NOBA (1 mg/mL in acetonitrile), the mix was added in 96 wells microplates (Falcon TM) and incubated at 37 °C for 60 min [63]. The absorbances were recorded at 425 nm on a Multiskan Sky Microplate Spectrophotometer from Thermo Scientific (Waltham, MA, USA). Wells containing only the buffer were used as a blank. All samples were assayed in triplicate. Additionally, indirect hemolysis method using agarose-erythrocyte-egg yolk gels [64] was also used to PLA_2_ activity evaluate, to them doses of recombinant toxin (25 and 50 µg) or five µg of *M. dumerilii* venom diluted in 16 µL of SS were added into of each well. The assay was performed by triplicate, and measurements of the hemolytic halo diameter of each sample were in millimeters (mm). The erythrocytes were donated by the “Clínica León XIII” blood bank of the Universidad de Antioquia.

### 5.12. Anticoagulant Activity

A total of 25 µg of His-rMdumPLA_2_ dissolved in 50 µL PBS (0.12 M NaCl, 0.04 M sodium phosphate, pH 7.2) were preincubated for 10 min at 37 °C with 200 µL of citrated human plasma obtained from the “Clínica León XIII” blood bank of The Universidad de Antioquia. After that, 100 µL of 0.25 M CaCl_2_ was added, and clotting times were recorded. PBS was used as a negative control. Both negative control and samples were assayed in triplicate.

### 5.13. Lethal Activity

To determine the lethality of recombinant toxin, a dose 100 µg/mouse (6 µg/g body weight) of His-rMdumPLA_2_ in 300 µL of SS was injected by the intraperitoneal (i.p.) route in two mice. A control group received SS alone. After, deaths were recorded within the following 24 h.

### 5.14. Antibodies Anti-His-rMdumPLA_2_ Production

One New Zealand white rabbit was immunized by subcutaneous route with a first dose (325 µg) of the recombinant His-rMdumPLA_2_ protein diluted in 600 µL of saline solution and emulsified with 600 µL of incomplete Freund’s adjuvant (IFA). Then, rabbit was boosted 21, 42, 63, 84, 105, 133 and 162 days with 488, 732, 1098, 1647, 1647, 1647 and 2470 µg of His-rMdumPLA_2_, respectively. The rabbit was bled on the first day (pre-immune) and after of first, third, fifth and seventh boosted.

### 5.15. Antibodies Anti-His-rMdumPLA_2_ Titers and Immunological Recognition in Serum by ELISA

For measuring the antibody titers, two assays by ELISA were performed: First, a tittering curve using different dilutions of the sera obtained from the bleedings, and second, an assay of the immunological recognition of His-rMdumPLA_2_ for specific rabbit serum using the dilution with the higher antibody titer. Firstly, bleedings were centrifuged at 6500× *g* for 10 min at 4 °C, and the supernatants (sera) were recovered. Afterward, several centrifugations were performed under the same conditions until the sera were clarified. A curve of different sera dilutions was assayed (1:10, 1:100, 1:1000, 1:5000, and 1:10,000), and two controls (serum pre-immune as negative control and *M. dumerilii* venom as positive control) were included. Next, a 96-well plate (Falcon TM) was covered with a 100 µL solution [1 µg/mL] of His-rMdumPLA_2_ or of complete *M. dumerilii* venom in coating buffer (0.1 M Tris, 0.15 M NaCl, pH 9.0) by overnight incubation. After five washes for 5 min each with wash buffer (0.05M Tris, 0.15M NaCl, 20 mM ZnCl_2_, 1mM MgCl_2_, pH 7.4), wells were blocked with 100 µL of blocking buffer (PBS containing 2% BSA), for 60 min, and decanted. Then, 100 µL of each dilution of rabbit immune serum, or a serum from a non-immunized rabbit, were identically diluted in PBS-BSA and added for 1.5 h. Subsequently, five washes with PBS for 5 min each were performed, followed by adding 100 µL of antirabbit immunoglobulins–Peroxidase conjugate (Sigma, Saint Louis, MO, USA), diluted 1:8000 in PBS-BSA, for 1.5 h. Finally, five washes for 5 min each with PBS were performed, and color was developed with 100 µL/well of o-Phenylenediamine (OPD) [2 mg/mL] in sodium citrate buffer (C_6_H_5_Na_3_O_7_-2H_2_O), pH 5.0. All samples were assessed in triplicate, and absorbances were recorded at 490 nm on a Multiskan Sky Microplate Spectrophotometer from Thermo Scientific (Waltham, MA, USA). Secondly, we followed the protocol above in another 96 wells microplate and used the same controls. Still, this time, 100 µL of the dilution 1:1000 of rabbit immune serum in PBS-BSA was added for 1.5 h, and the rest of the steps were followed, as mentioned above.

### 5.16. Anti-His-rMdumPLA_2_-IgG Purification

To obtain the IgG of serum anti-His-rMdumPLA_2_, fractionation with caprylic acid (Sigma, Saint Louis, MO, USA) was performed [65]. For this, small amounts of caprylic acid were added to each serum until reaching 6% of the total volume of the serum. The solution was kept under constant and vigorous stirring for 1 h. The precipitated was separated by centrifugation at 4500× *g* for 5 min, and the supernatant was dialyzed on Fisherbrand cellulose membranes (3500 MWCO) with PBS and distilled water for two days. Finally, it was lyophilized in a LABCONCO (Kansas, MO, USA) lyophilizer using a range of temperature changes during processing (−40 °C to 10 °C) and stored at −20 °C.

### 5.17. Cross Immunological Recognition by Anti-His-rMdumPLA_2_-Serum and Anti-His-rMdumPLA_2_-IgG by ELISA

Following the description of “Section 5.15.” of Materials and methods, we performed an assay of cross immunological recognition by anti-His-rMdumPLA_2_-serum and His-rMdumPLA_2_-IgG in the presence of: (1) *M. dumerilii* venom (V-Mdum). (2) Two fractions (F24 and F25) close to MdumPLA_2_. These fractions were isolated from *M. dumerilii* venom by RP-HPLC and belong to the region of PLA_2_s obtained from its proteome [9]. (3) MmipPLA_2_, the most abundant PLA_2_ from *M. mipartitus* venom [8].

### 5.18. Neutralizing Ability of Anti-His-rMdumPLA_2_-IgG

*PLA_2_ activity***:** 3 mg anti-His-rMdumPLA_2_-IgG was mixed with 25 µg His-rMdumPLA_2_, the mixtures were incubated for 30 min to 37 °C and added to plates (Corning^®^, ref 3098) of agarose-erythrocyte-egg yolk gels and incubated 20 h. A total of 5 µg of *M. dumerilii* venom was used as a positive control. All assays were performed in triplicate, and measurements of the hemolytic halo diameter of each sample were expressed in millimeters (mm). The assays were performed in triplicate.

*Myotoxic activity:* 3 mg of anti-His-rMdumPLA_2_-IgG was mixed with 5 µg of *M. dumerilii* venom and diluted in 100 µL saline solution, incubated for 30 min to 37 °C, and injected intramuscular (i.m.) route to groups of three mice in the muscle gastrocnemius. A group with whole venom was used as a control. The CK activity was evaluated as previously described.

*Lethal effect:* 5 mg anti-His-rMdumPLA_2_-IgG was mixed with 1.5 LD_50_ (30 µg/mouse) of *M. dumerilii* venom in 300 µL of saline solution, incubated for 30 min at 37 °C and injected intraperitoneal (i.p.) route to two mice. A group with whole venom was used as a control. After 24 h later the number of deaths was recorded.

### 5.19. Statistical Analysis

The PLA_2_ activity, myotoxicity, anticoagulation, and edema induction experiments were performed in triplicate and the results were expressed as mean ± SD (*n* = 3). The immunological cross-recognition experiment was carried out by ELISA test. In the PLA_2_ activity and myotoxicity experiments, as in the ELISA test, the significant differences between the His-rMdumPLA_2_ treatments and the controls were determined using the one-way ANOVA test, followed by the Bonferroni post-test. In the same way, the neutralization experiments were analyzed using a two-way ANOVA test. In the edema induction activity experiment, the significant differences between the His-rMdumPLA_2_ treatments and the controls were determined using the unpaired t-test. In all cases, differences were considered significant for *p* < 0.05.

## Data Availability

Not applicable.
